# The Effect of Pre-Emptive Analgesia on the Postoperative Pain in Pediatric Otolaryngology: A Randomized, Controlled Trial

**DOI:** 10.3390/jcm11102713

**Published:** 2022-05-11

**Authors:** Jakub Zieliński, Monika Morawska-Kochman, Krzysztof Dudek, Michał Czapla, Tomasz Zatoński

**Affiliations:** 1 Department of Otolaryngology, Head and Neck Surgery, Wroclaw Medical University, 50-556 Wroclaw, Poland; jakub.zielinski@umw.edu.pl (J.Z.); monika.morawska-kochman@umw.edu.pl (M.M.-K.); tomasz.zatonski@umw.edu.pl (T.Z.); 2 Faculty of Mechanical Engineering, Wroclaw University of Technology, 50-231 Wroclaw, Poland; krzysztof.dudek@pwr.edu.pl; 3 Laboratory for Experimental Medicine and Innovative Technologies, Department of Emergency Medical Service, Wroclaw Medical University, 51-616 Wroclaw, Poland; 4 Group of Research in Care (GRUPAC), Faculty of Nursing, University of La Rioja, 26006 Logrono, Spain

**Keywords:** pain, postoperative pain, children, pain management, pre-emptive analgesia, adenoidectomy, tonsillectomy

## Abstract

The aim of this randomized, controlled trial was to determine whether children undergoing otolaryngological procedures (adenoidectomy, adenotonsillotomy, or tonsillectomy) benefit from pre-emptive analgesia in the postoperative period. Methods: Fifty-five children were assessed for eligibility for the research. Four children refused to participate during the first stage of the study, leaving fifty-one (*n* = 51) to be randomly assigned either to receive pre-emptive analgesic acetaminophen (15 mg/kg; *n* = 26) or a placebo (*n* = 25) in addition to midazolam (0.5 mg/kg) as premedication. All children were anesthetized with sevoflurane, propofol (2–4 mg/kg), and fentanyl (2 mcg/kg). Postoperative pain was assessed using the Visual Analogue Scale (VAS), the Wong–Baker Faces Pain Rating Scale, and the Face, Legs, Activity, Cry, and Consolability (FLACC) scale. The postoperative pain was measured 1, 2, 4, and 6 h after the surgery. Results: The clinical trial reported a statistically significant correlation between administering pre-emptive analgesia (acetaminophen) and reducing pain in children after otolaryngological procedures compared to placebo. The ratio of boys to girls and age were similar among the groups (*p* > 0.05), so the groups of children were not divided by gender or age. Conclusions: Standard pre-emptive analgesia reduced the severity of pain in the postoperative period after otolaryngological procedures in children. Acetaminophen given before surgery reduces postoperative pain in children undergoing otolaryngological procedures.

## 1. Introduction

Adenoidectomy, tonsillectomy, or adenotonsillotomy are some of the most commonly performed surgeries in children all around the world. Tonsillectomy consists of the complete removal of the tonsils [1], but it is very often performed together with adenoidectomy for the treatment of obstructive sleep apnea, sleep-disordered breathing, or chronic tonsillitis [1,2,3]. It was previously reported that tonsillectomy is an effective treatment tool for obstructive sleep apnea in children [4,5]. The removal of the tonsils triggers inflammatory cascades that improve healing but also leaves an open wound in the oropharynx, which exposes the glossopharyngeal and trigeminal nerves [6]. This combination of factors causes a postoperative wound that is susceptible to mechanical trauma when swallowing. Therefore, this procedure is associated with severe postoperative pain [7]. The pain after tonsil removal begins with local tissue damage and does not subside completely until the affected lesion is covered with a mucous membrane. Common complications following tonsillectomy include nausea and vomiting, hemorrhage, dehydration, and pain [8].

Postoperative analgesia in pediatric patients undergoing tonsil surgery is a subject that has been widely discussed by ENT (ear, nose, and throat) specialists for years. The basic goals for surgical centers are painless hospitalization with a focus on a short hospital stay, an early home discharge and return to normal activities, and the prevention of re-hospitalization due to complications. Appropriate analgesic therapy requires standard protocols. Increasing awareness and effort to improve the pain management peri- and postoperatively in children have resulted in the development of guidelines in 2018 by the European Society for Paediatric Anaesthesiology (ESPA) Pain Committee [9]. These guidelines were primarily addressed to the European continent, but they can also be used in other countries around the world, according to the availability of medicines, national recommendations, and drug registration rules. In addition, the research into postoperative pain management led to the concept of pre-emptive analgesia [10]. Pre-emptive analgesia is a method that is initiated before the pain stimulus occurs, namely, before the surgery begins, and is continued during the procedure to minimize the physiological consequences of nociceptive transmission induced by the surgery. As a result of this “protective” effect, it may be more effective than similar analgesic treatment initiated after surgery. Pre-emptive analgesia can be used to prevent a pain signal from the surgical wound starting from the very first skin incision. These findings have been confirmed in previously published studies on pre-emptive analgesia in different surgical conditions [11,12,13]. The aim of this double-blinded, randomized, placebo-controlled trial was to determine whether children undergoing otolaryngological procedures (adenoidectomy, tonsillectomy, or adenotonsillectomy) benefit from pre-emptive analgesia in the postoperative period.

## 2. Materials and Methods

Approval was obtained from the Ethical Committee of the Medical University of Wrocław to perform the study under the number KB–459/2018. The authors confirm that all procedures comprising this research conformed to ethical standards and institutional guidelines for human experimentation. The study was registered in the ISRCTN registry under the number ISRCTN77862744 and was performed from July 2019 to February 2022. Trial registration: 26 April 2021—retrospectively registered.

The primary outcome of the study was to determine the effect of pre-emptive analgesia on postoperative pain in pediatric otolaryngology. The secondary outcome provides the correlation between pain scores measured with the Visual Analogue Scale (VAS), the Wong–Baker Faces Pain Rating Scale (WB), and the Face, Legs, Activity, Cry, and Consolability (FLACC) scale after surgery. Assuming a probability of first type error (alpha) at *p* < 0.05 and a test power (1—beta) > 0.80 and the expected treatment effect: for the significance test for related variables, the required group size is N = 45.

### 2.1. Research Participants

**Figure 1:**
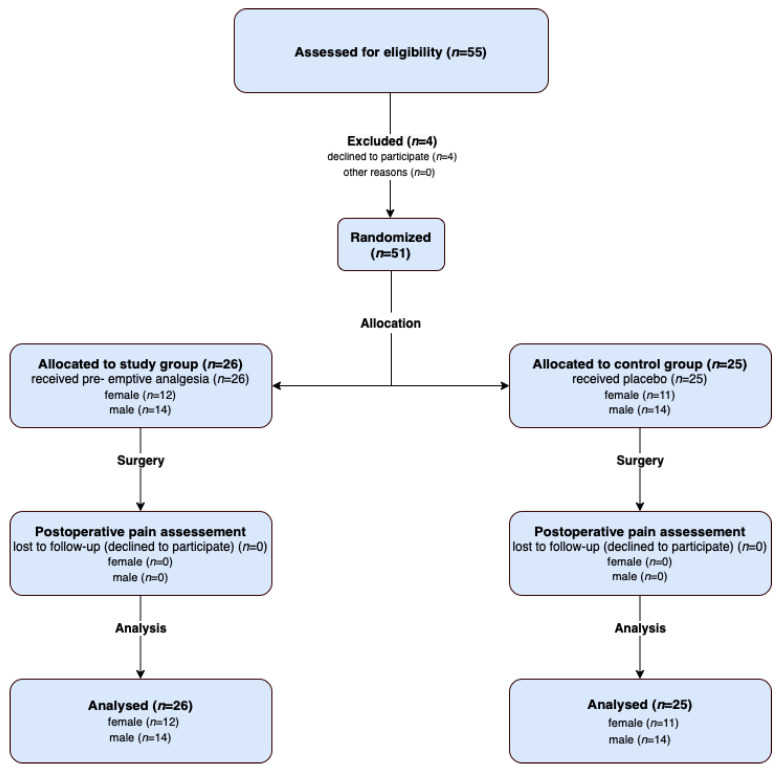
Flow diagram presenting the research participants.

The clinical trial initially included a total of 55 children from the Department of Otolaryngology of the University Clinical Hospital in Wrocław. Four children who refused to participate at the very beginning of the study, with a subsequent lack of parental formal consent, were excluded, resulting in an overall number of 51 children (Figure 1). The inclusion criteria were an age of 3–15 years and the written informed consent from the parents (or legal guardians). The exclusion criteria were intellectual disability; major coexisting diseases; allergy to acetaminophen, dexamethasone, or nalbuphine; and pain prior to surgery.

### 2.2. Randomization and Blinding

To randomly allocate a patient to a study or control group, permuted block randomization was used. The patient, parent, anesthetist, and surgeon were all blinded to the study. Only the nurse delivering the premedication to the child was not blinded. Once the child received their premedication, the group allocation sheet was concealed in an envelope. The exact dose of postoperative analgesics was prescribed every 6 h, based on the child’s weight.

### 2.3. Pain Assessment

Postoperative pain was assessed using the Visual Analogue Scale (VAS), the Wong–Baker Faces Pain Rating Scale (WB), and the Face, Legs, Activity, Cry, and Consolability (FLACC) scale. The FLAAC scale [14] is an assessment tool that is used to quantify pain on a score from 0 to 10 using five categories: facial expression, legs, activity, cry, and consolability. The observation should last 2–5 min. Each parameter is evaluated on a scale from 0 to 2; the total score is interpreted as follows: 0 = Relaxed and comfortable, 1–3 = Mild discomfort, 4–6 = Moderate pain, 7–10 = Severe discomfort/pain. A score of more than 3 points suggests the need for analgesics. The Wong–Baker FACES Pain Rating Scale [15] was the second assessment tool used during the postoperative period. It consists of a series of faces from a 0-value happy face—which represents a lack of pain—to a 10-value crying face, which suggests the worst possible pain. The patient chooses the face that best reflects their current pain level. The VAS [16] is a line that is often 10 cm long with the markings “1” and “10” at the ends. The “1” represents no pain or discomfort, and “10” indicates very strong pain. The patient is asked to mark a line on the scale to express the intensity of their pain.

### 2.4. Research Protocol including Anesthesia Protocol

Fifty-one children were randomized to receive either pre-emptive analgesic acetaminophen (Pedicetamol, Laboratorios ERN, Barcelona, Spain) at the dose of 15 mg/kg (*n* = 26) or a placebo (*n* = 25), in addition to midazolam (Midanium, WZF Polfa S.A., Warsaw, Poland) at the dose of 0.5 mg/kg (N = 51) as premedication. Only the nurse delivering the premedication to the child was not blinded; the group allocation sheet was concealed in an envelope after the premedication liquid was administered to the child orally. The research documents were stored in a locker at the hospital, which only the investigators had access to.

The premedication mixture with acetaminophen and midazolam was red and strawberry-flavored. To assure that the control group was given a liquid that was the same weight and color, concentrated strawberry juice was added to the midazolam. Approximately 30–45 min after the premedication was given, the child was transported to the operating room.

The induction of anesthesia was performed with propofol (Propofol 1% MCT/LCT, Fresenius Kabi, Bad Homburg, Germany) at the dose of 2–4 mg/kg iv, sevoflurane (Sevoflurane Baxter, Baxter Polska, Warsaw, Poland) as inhalation agent and fentanyl (Fentanyl WZF, WZF Polfa S.A., Warsaw, Poland) at the dose of 2 mcg/kg iv. Sevoflurane was used to maintain anesthesia. At the end of surgical procedures, all patients received intravenous dexamethasone (Dexaven, Bausch Health Ireland Limited, Dublin, Ireland) at the dose of 0.2 mg/kg and nalbuphine (Nalpain, G.L. Pharma GmbH, Vienna, Austria) at the dose of 0.2 mg/kg as standard perioperative analgesia. Postoperatively the patients were transferred to the postop room and then to the otolaryngology ward, which took approximately 45 min.

Postoperative pain was assessed using the Wong–Baker Faces Pain Rating Scale, the VAS, and the FLACC scale. The postoperative pain was measured 1, 2, 4, and 6 h after the surgery. The children were asked to score their pain on the VAS and Wong–Baker scale, while the FLACC scale was used by the research team to assess pain. During the evaluation, the research team and the parent or guardian were present. Only throat pain was included in the pain score analysis. All children in this study received postoperative analgesia. The patients were administered intravenous acetaminophen (Paracetamol B. Braun, B. Braun Melsungen AG, Melsungen, Germany) at the dose of 15 mg/kg every 6 h after the previous dose was given. In case of severe pain, additional intravenous metamizole (Pyralgin, Polpharma S.A, Starogard Gdanski, Poland) at the dose of 15 mg/kg was administered.

### 2.5. Statistical Analysis

Statistical analysis was performed using the STATISTICA v.13.3 software (TIBCO Software Inc., Palo Alto, CA, USA). Qualitative variables are presented in the contingency tables as numbers and proportions. The independence of the two qualitative variables was verified using the Chi-square test. For quantitative variables, mean values, median standard deviations, quartile ranges, and extreme values were calculated. The Shapiro–Wilk test was used to assess the normality of the distribution. As the empirical distributions deviated from the normal distribution, non-parametric tests were used in further analysis. The Mann–Whitney U test was used to estimate the statistical significance of the difference between independent groups. The relationship between pain scales was checked by calculating Spearman’s Rho correlation coefficient. The values of the intra-class correlation coefficients (ICC) and Bland–Altman plots were used to assess the compliance of the results of pain level measurements on the WB, VAS, and FLACC scales. The results were considered statistically significant at *p* < 0.05.

## 3. Results

### 3.1. Demographic Data

Data were collected on 51 children (23 girls and 28 boys). The children were divided into two groups. The first one consisted of twenty-six children (51%) who received acetaminophen prior to surgery. The second one consisted of twenty-five children (49%) who received a placebo. The mean age was 5.6 years (SD = 2.8; range: 2–15) (Table 1). Both groups were properly matched, and there were no statistically significant differences between the two research samples: the gender and age ratios were similar. There was no statistically significant correlation between the use of pre-emptive analgesia and gender or age (Table 2).

### 3.2. Outcomes

The study showed a statistically significant difference in postoperative pain between the two study groups. The level of pain in children after otolaryngological procedures, who received acetaminophen as pre-emptive analgesia, was significantly lower compared to those who received placebo, when using Wong–Baker Faces Pain Rating Scale and the VAS, except for the second hour after surgery (Table 3). However, the differences in pain scores on the FLACC scale are not statistically significant (*p* > 0.05). There was no need to provide rescue analgesia for any patients during this trial.

A statistically significant correlation was found between pain scores measured by WB, VAS, and FLACC scales at the first, second, fourth, and sixth hours after surgery (Table 4).

**Figure 2:**
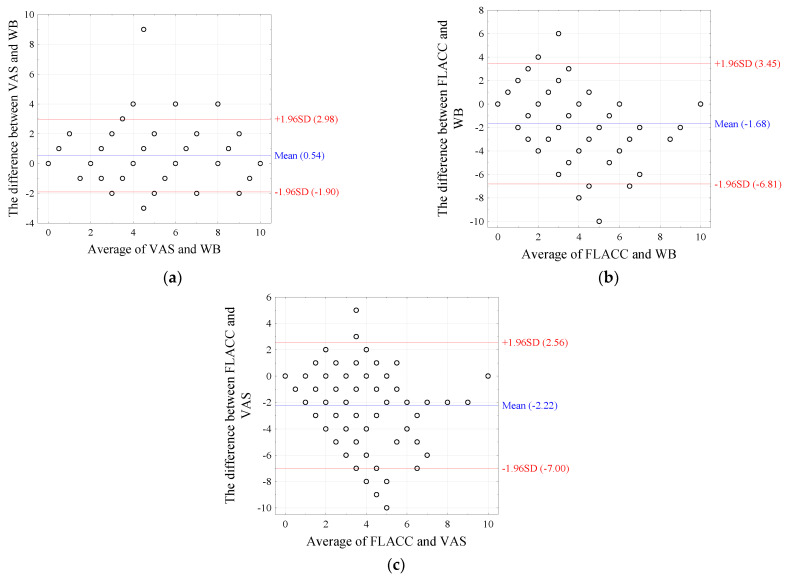
Bland–Altman charts comparing pain levels as assessed by WB, VAS, and FLACC.

The highest rate of agreement between pain level assessment scales was between the WB and VAS scales (Table 5). The Bland–Altman index, i.e., the percentage of ratings not falling within the 95% concordance range for the mean difference, was 9.8% for VAS vs. FLACC, and for WB vs. FLACC ratings, it was 11.8% (Figure 2).

## 4. Discussion

Postoperative pain management in children is challenging, especially after tonsillectomy, which is a painful procedure for most children—much more painful than an adenoidectomy [17]. In recent years, several tools have been applied preoperatively to reduce postoperative pain, including injections of local anesthetics prior to surgery, the application of fibrin glue, pre-emptive peritonsillar infiltration of magnesium sulfate or magnesium, the administration of steroids [18], different surgical approach, and the administration of oral analgesics. Each method has its own benefits, but none has consistently proven to have a statistically significant reduction in postoperative pain in children. Peritonsillar infiltration with ropivacaine [19] or magnesium sulfate [20] did not provide any major postoperative analgesic effect in children after adenotonsillectomy. Additionally, there was no significant beneficial effect of fibrin glue in post-tonsillectomy pain control [21]. In contrast, pre-emptive peritonsillar dexamethasone infiltration reduced post-tonsillectomy pain better than levobupivacaine [22]. Another study did not confirm the hypothesis that tonsillectomy performed with the coblator device would result in lower levels of postoperative pain [23].

This study showed that acetaminophen administered as a part of oral premedication effectively reduced the level of postoperative pain in children and thus demonstrated the effectiveness of pre-emptive analgesia. In order to exclude the influence of other potentially analgesic agents in this study, a uniform anesthetic protocol common to all participating children was established. The use of local anesthesia or other drugs outside the protocol was excluded. The surgery times were similar, and all procedures were performed without complications.

The selection of acetaminophen, among other analgetic drugs commonly used in pain management in the otolaryngological ward, such as ibuprofen, opioids, and steroids for pre-emptive analgesia, was due to its limited side-effect profile. Acetaminophen has been shown to be very safe for use in children [24]. It is an effective analgesic in the postoperative period [25]. Acetaminophen is metabolized in the liver by CYP2E1, and toxicity and mortality occur only when taken in massive quantities [26]. Acetaminophen is well-tolerated and has an adequate analgesic effect in children after tonsillectomy [27]. As a nonsteroidal anti-inflammatory drug (NSAID), ibuprofen remains controversial because of its nonselective inhibition of cyclooxygenase (COX), resulting in limited production of proinflammatory cytokines, and because it leads to blocking the formation of thromboxane (part of platelet aggregation) [28], potentially resulting in an increased risk of bleeding. Nevertheless, several systematic reviews have not substantiated a statistically significant difference in the frequency of bleeding postoperatively when comparing NSAIDs to other analgesics [29,30]. Another review [31], which included only studies that used ibuprofen and looked at the bleeding rates for the entire postoperative period, found a statistically significant 35% increase in post-tonsillectomy hemorrhage with ibuprofen. In addition, NSAIDs manage pain similarly to opioids and other analgesics [32,33], and they reduce the incidence of postoperative vomiting [34].

Another analgesic drug considered was dexamethasone steroid. A single intraoperative dose of intravenous dexamethasone reduces postoperative nausea and pain [35]. It is recommended by the American Academy of Otolaryngology—Head and Neck Surgery, which includes steroids in its clinical practice guideline. Multiple studies have concluded that there is no increased risk of postoperative bleeding associated with a single perioperative dose of steroids [36,37,38].

Opioids were excluded because special precautions should be taken when prescribing opioids in patients with obstructive sleep apnea due to the increased risk of ventilation disorders in the postoperative period [39]. However, a systematic review [40] concluded that there was not any relevant efficacy advantage between opioids, steroids, ibuprofen, and acetaminophen. For the above discussed reasons, acetaminophen was chosen for this study.

The idea of preemptive analgesia before tonsillectomy is not new. Still, our work advantage and novelty are the use of several pain scales, including those designed especially for children. In work published by El-Fattah and Ramzy [41], the parent-specific pain rating scale was used. Although the caregiver’s assessment of pain may be subjective, the effectiveness of pre-emptive analgesia for pain reduction after surgeries in children was demonstrated, as in our study.

Assessing the pain children experience can be problematic because children are unwilling or unable to verbalize. Current standard pain assessment tools mostly rely on self-reporting and behavioral analysis of children, and a wide range of studies have developed methods to accurately assess the intensity of pain in children in the postoperative period. The pain scales most often used in the literature are the FLACC scale, the Wong–Baker FACES Pain Rating Scale, and the VAS [42]. 

In this clinical trial, the research team concluded that postoperative pain in children should be assessed using the most popular and widely validated scales. Explaining the Wong–Baker and the VAS to the children in the preoperative period improved their understanding and informed pain scale marking in the postoperative period. In addition, children were asked to locate the site of pain in postoperative assessment—which was throat pain, excluding the intravenous cannula or other reasons. It was easier for both the patient and the research team and led to a better relationship with the child in the postoperative period.

A standard anesthesia protocol was used in this study, including sevoflurane, intravenous propofol (2–4 mg/kg), and fentanyl (2 mcg/kg) induction. During the surgery, all patients received a single intravenous dose of dexamethasone (0.2 mg/kg) and nalbuphine (0.2 mg/kg) as standard perioperative analgesia. Postoperatively, the patient was transferred to the postop room and then to the otolaryngology ward, which took approximately 45 min. Sevoflurane is the most popular gas induction agent; it is tolerated well by patients and is known for its beneficial pharmacokinetic and pharmacodynamic properties [43]. However, it is associated with an increased risk of emergence agitation, which presents as pain-like signs, including crying and moaning, confusion, disorientation, and incoherence [44]. Emergence agitation can last up to 45 min and can occur independently of pain [45]. Therefore, postoperative pain was assessed 1, 2, 4, and 6 h after surgery. In addition, it has been proven that administering fentanyl around the end of surgery reduces the incidence of emergence agitation in children undergoing general anesthesia [46]. Furthermore, propofol also improves analgesia, produces sedation, and results in immediate recovery with hemodynamic stabilization and minimal respiratory depression [47].

### Limitations

The limitations of the present study rest on the fact that it is a single-center study with a small number of patients. The reason for this was the fact that the time implementation of the study coincided with the COVID-19 pandemic, and thus, the number of planned procedures performed during that period was limited. The observation and pain recording period was limited to 6 h after the surgery; therefore, it would be worth extending it to 24 h in future studies. This would give an answer to the question of whether pre-emptive analgesia influences the pain level at a later period than only a few hours after the surgery. It is therefore uncertain whether the results can be replicated in other larger centers, with variable experience and standardization in their practices.

## 5. Conclusions

The clinical trial reported a statistically significant correlation between administering pre-emptive analgesia (acetaminophen) and reducing pain in children after otolaryngological procedures compared to placebo. The majority of children in both study groups did not experience severe postoperative pain regardless of whether or not they received acetaminophen before the procedure. However, the positive effect of pre-emptive analgesia was statistically significant in reducing pain to an absolute minimum. For this reason, it seems that pre-emptive analgesia should be routinely used in children undergoing otolaryngological procedures.

## Figures and Tables

**Table 1:** Characteristics of the studied children.

Variable	Statistics
Gender	N (%)
Female	23 (45.1%)
Male	28 (54.9%)
Age (years):	
Mean ± SD	5.6 ± 2.8
Me (Q1–Q3)	5 (4–7)
Min–Max	2–15
Pre-emptive	
Yes	26 (51.0%)
No	25 (49.0%)

Abbreviations: Me—median.

**Table 2:** Characteristics of children in groups that differ in pain prevention.

Variable	Pre-Emptive Analgesia		*p*-Value
	Yes N = 26	No N = 25	
Gender:			0.877
Female, *n* (%)	12 (46.2)	11 (44.0)	
Male, *n* (%)	14 (53.8)	14 (56.0	
Age, years			0.258
Mean ± SD	6.0 ± 2.7	5.2 ± 2.8	
Me (Q1–Q3)	5 (4–8)	5 (3–6)	
Min–Max	3–13	2–15	
Body weight (kg)			0.172
Mean ± SD	28.0 ± 14.0	23.1 ± 11.1	
Me (Q1–Q3)	23 (20–29)	20 (17–24)	
Min–Max	15–68	13–63	
Height (cm)			0.113
Mean ± SD	123 ± 18	115 ± 17	
Me (Q1–Q3)	120 (107–130)	111 (104–126)	
Min–Max	102–160	85–160	
BMI (kg/m^2^)			0.449
Mean ± SD	17.6 ± 3.6	16.9 ± 3.4	
Me (Q1–Q3)	16.4 (15.3–18.8)	15.9 (14.6–19.9)	
Min–Max	13.3–29.4	12.4–24.6	

Abbreviations: Me—median; *p*-value, *p*—level of significance; *n*—number of participants, BMI—body mass index.

**Table 3:** Assessment of perceived pain in groups of children differing in pain prophylaxis.

Pain Measurement Scale	Pre-Emptive Analgesia		*p*-Value
	Yes N = 26	No N = 25	
WB 1 h	2 ((0–2)	4 (2–8)	0.001
WB 2 h	2 (0–4)	2 (0–4)	0.401
WB 4 h	2 (0–4)	4 (2–6)	0.002
WB 6 h	0 (0–2)	4 (2–4)	0.003
VAS 1 h	2.5 (1–4)	6 (3–8)	0.001
VAS 2 h	2 (1–5)	3 (1–5)	0.839
VAS 4 h	2 (1–3)	4 (3–6)	0.001
VAS 6 h	1 (1–2)	4 (2–5)	0.002
FLACC 1 h	0 (0–4)	2 (0–4)	0.289
FLACC 2 h	0 (0–2)	0 (0–2)	0.847
FLACC 4 h	0 (0–1)	0 (0–1)	0.779
FLACC 6 h	0 (0–0)	0 (0–0)	1.000

Abbreviations: *n*—number of participants; *p*-value, *p*—level of significance; WB—Wong–Baker Faces Pain Rating Scale; VAS—Visual Analogue Scale (VAS); FLACC—Face, Legs, Activity, Cry, and Consolability Scale.

**Table 4:** Rank correlation coefficients (Spearman’s Rho) between pain scales.

		WB	VAS	FLACC
1st hour	WB	×	0.854 ***	0.474 ***
	VAS		×	0.528 ***
	FLACC			×
2nd hour	WB	×	0.894 ***	0.318 *
	VAS		×	0.328 *
	FLACC			×
4th hour	WB	×	0.956***	0.323 *
	VAS		×	0.327 *
	FLACC			×
6th hour	WB	×	0.915 ***	0.515 ***
	VAS		×	0.511 ***
	FLACC			×

Abbreviations: WB—Wong–Baker Faces Pain Rating Scale; VAS—Visual Analogue Scale (VAS); FLACC—Face, Legs, Activity, Cry, and Consolability Scale. * *p* < 0.05; *** *p* < 0.001.

**Table 5:** The values of the intra-class correlation coefficients (ICC) assessing the compliance of the results of pain level measurements on the WB, VAS, and FLACC scales.

Patients	VAS vs. FLACC	WB vs. FLACC	VAS vs. WB
All	0.287	0.324	0.876
Pre-emptive	0.364	0.457	0.851
No pre-emptive	0.249	0.258	0.860

Abbreviations: WB—Wong–Baker Faces Pain Rating Scale; VAS—Visual Analogue Scale (VAS); FLACC—Face, Legs, Activity, Cry, and Consolability Scale.

## Data Availability

The data can be accessed by contacting the corresponding author.
